# Can real-world measures of activity, sleep, and cardiorespiratory function stratify Sjogren’s disease participants with persistent fatigue? Insights from the BRC tools study

**DOI:** 10.1016/j.ero.2025.11.004

**Published:** 2025-11-30

**Authors:** Chloe Hinchliffe, Bing Zhai, Victoria Macrae, Jade Walton, Silvia Del Din, Wan-Fai Ng

**Affiliations:** 1 Translational and Clinical Research Institute, Newcastle University, Newcastle upon Tyne, UK; 2 Computer and Information Science, Northumbria University, Newcastle upon Tyne, UK; 3 National Institute for Health and Care Research Newcastle Biomedical Research Centre, Newcastle upon Tyne Hospitals NHS Foundation Trust and Newcastle University, Newcastle upon Tyne, UK; 4 National Institute for Health and Care Research Newcastle Clinical Research Facility, Newcastle upon Tyne Hospitals National Health Service Foundation Trust and Newcastle University, Newcastle upon Tyne, UK; 5 Department of Rheumatology, Cambridge University Hospitals National Health Service Foundation Trust, Cambridge, UK; 6 Health Research Board Clinical Research Facility, University College Cork, Cork, Ireland

## Abstract

**Objectives:**

Fatigue is a common and debilitating symptom for many people with Sjogren’s disease (SjD). A major challenge in research with fatigue is its highly subjective nature, and unlike subjective measures of sleep and activity, no previous works have found reliable objective measures for identifying fatigue. The current analysis explored if categorising participants based on the patterns of their self-reported fatigue, rather than overall severity, would reveal more group differences in real-world measures of activity, sleep, and cardiorespiratory function estimated by digital wearables.

**Methods:**

In total, 97 participants with SjD wore a VitalPatch chest-worn sensor and ActiGraph wrist-worn sensor for up to two 7-day periods while self-assessing their fatigue severity up to 4 times daily. These devices estimated 31 measures relating to activity, sleep, and cardiorespiratory function. Participants were grouped based on the persistency of their fatigue, and for comparison, a more conventional approach grouped participants based on their overall fatigue.

**Results:**

Between-group differences for both approaches showed that those with more fatigue had reduced activity intensity, poorer sleep quality, and a higher heart rate. But, separating participants by fatigue persistency, compared with overall fatigue, returned more statistically significant (*P* < .05) parameters, 10 compared with 5, indicating clearer relationships when this approach is taken.

**Conclusions:**

Isolating participants with higher lower-variable fatigue revealed more between-group differences than that with simply isolating those with higher overall fatigue. This alternative strategy demonstrates that digital wearables do have the potential to identify fatigue in participants with SjD. Thus, contributing towards objective, continuous estimates of fatigue in chronic diseases.

**WHAT IS ALREADY KNOWN ON THIS TOPIC**

• Fatigue is a common and debilitating symptom for many people with Sjogren’s disease, and in part, due to highly subjective nature of fatigue, no objective biomarker has been identified.

**WHAT THIS STUDY ADDS**

• The current analysis proposes that categorising participants based on the persistence of their fatigue, as opposed to overall severity, may be more effective when analysing objective measures of physiology collected by digital wearables.

**HOW THIS STUDY MIGHT AFFECT RESEARCH, PRACTICE, OR POLICY**

• Separating participants based on fatigue persistence revealed more differences with objective measures of activity intensity, heart rate, and sleep quality.

Alt-text: Unlabelled box

## INTRODUCTION

Sjogren’s disease (SjD) is an immune-mediated inflammatory disorder that leads to dry mouth, dry eyes, and musculoskeletal pain [1]. Like many individuals with a chronic disease, these patients can experience debilitating fatigue that substantially impacts their quality of life [2]. For example, impaired stamina and fatigue, as opposed to dryness, have been shown to be linked with early retirement, and those with severe fatigue who remained at work showed an increase in physician visits [3]. People with SjD have described their fatigue as horrible, leading to an increase in their stress [4]. For some, fatigue onset is sudden and unpredictable, whereas others are able to anticipate and plan around its fluctuations [4]. Fatigue is also highly prevalent in SjD: 67% reported clinically important levels of fatigue [2], and SjD is associated with increased fatigue compared with healthy individuals [5].

To develop solutions to this important symptom, reliable estimates of fatigue severity is necessary for evaluating intervention efficacy. Currently, assessments of fatigue rely on patient-reported outcomes (PROs), which consist of questionnaires and diaries designed to record how the patient is feeling. However, PROs are subjective, susceptible to recall bias [6], and only provide a snapshot view of fatigue. Wearable devices, however, can provide objective, reliable, and continuous estimates of human behaviours and physiology in free-living environments. Physical activity, sleep, and cardiorespiratory function are essential components of health and may provide objective evidence of fatigue [7] and could therefore aid in patient feedback and therapeutic development to mitigate these symptoms.

Most of the literature exploring the relationships between fatigue and activity or sleep in SjD has used subjective rather than objective measures. For activity, subjective measures collected through questionnaires and diaries were inconsistent in establishing an association with fatigue; 2 studies found less activity was associated with higher general [8], physical [8,9], or mental fatigue [9], while 3 others found no association with mental [8] or general fatigue [10,11]. One study used an objective measure of habitual activity with a hip-worn ActiGraph sensor [12]. In total, 29 participants with SjD wore the device to collect at least 10 hours of valid activity recordings per day for at least 5 days, from which the authors estimated sedentary time, light activity counts and moderate-to-vigorous physical activity counts. This study found no correlation between these physical activity measures and patient-reported fatigue estimated with FACIT-F [13] and visual analogue scale (VAS) [14].

Similarly, in sleep, studies using subjective measures were inconsistent in reporting an association with fatigue. Eight studies found an association between subjectively assessed sleep quality measures and fatigue in SjD [[15], [16], [17], [18], [19], [20], [21], [22]], while 2 studies found no relationship [11,18]. However, 3 studies that used objective assessments of sleep found no relationship with fatigue in SjD. These studies included 25 [22], 28 [23], and 50 [21] participants with SjD; estimated sleep quality using in-laboratory polysomnography (PSG) [23] and actigraphy (a wrist-worn accelerometer) [21,22]; and estimated fatigue with FACIT-F [23], VAS [21,22], Profile of Fatigue and Discomfort–Sicca Symptoms Inventory (short form) [21], Likert scale [22], and Medical Outcomes Study: Short Form-36 [22]. In addition, only 1 study was found to explore measures of cardiorespiratory rate in SjD, which found that increased heart rate (HR) during a supine test was found to be associated with increased fatigue [24].

Thus, the evidence available to date does not reveal a clear relationship between measures of activity, sleep, and fatigue severity. This is possibly due to the challenge of accurately and precisely self-rating one’s own sleep quality and/or activity intensity. Factors such as recall bias, subjective perception, and variability can impact the PRO–that is, if one felt very sleepy for 30 minutes in morning but felt very awake for the rest of the day, how one rates sleepiness for that day is likely to be varied between individuals. Since the literature indicates a discrepancy in how participants perceive their activity, sleep, and fatigue levels, it is possible that the statistical approach of comparing these measures to contribute to the inconsistency of these studies’ findings. Currently, the literature compares fatigue to measures of sleep and activity using correlations [[8], [9], [10], [11], [12],[15], [16], [17], [18], [19], [20], [21], [22], [23]], low vs high fatigue group comparison [17], or low vs high measure (sleep quality or activity levels) group comparison [10,11]. In this study, we propose an alternative strategy to analysing fatigue PROs: persistent fatigue. For example, those who experience consistently high fatigue may have a different perception and/or coping mechanisms for their fatigue symptoms, since they may have developed adaptations compared with those who experience more variable, unpredictable fatigue.

Accordingly, we hypothesise that digital measures of physiological parameters differ between patients with SjD and persistent fatigue and those with lower, more variable fatigue. This study aims to use the data from The Newcastle Biomedical Research Centre (BRC) Tools study [25] to explore the relationships between objective real-world measures of activity, cardiorespiratory function, and sleep and self-reported fatigue by determining the pattern of their fatigue and to compare this approach with the more conventional approach of inspecting overall fatigue.

## METHODS

### Data

In total, 100 participants with SjD were recruited from the BRC Tools study (Integrated Research Application System (IRAS): 247550), which was approved by the North East–Newcastle and North Tyneside 1 Research Ethics committee (19/NE/0002). Inclusion criteria include aged 18 years or older, fulfilled the American European Consensus Group classification criteria for SjD, and were willing and able to provide consent and participate. Exclusion criteria ensured the participants were not depressed or anxious, experiencing chronic pain or severe skin allergies, or presenting with cognitive impairment. Full details can be found in the Supplementary Materials.

Participants wore 2 sensors, on the chest and wrist, for two 7-day continuous periods while at home, spaced 6 to 8 weeks apart (although this was not always possible due to the COVID-19 pandemic). The chest sensor was VitalConnect’s VitalPatch [26], a 12-cm device containing a single-lead electrocardiogram (ECG), a triaxial accelerometer, and a thermistor. The sampling rates for the ECG and accelerometer were 125 and 62.5 Hz, respectively. The wrist-worn sensor was an ActiGraph GT9X Link [27]. This was an inertial measurement unit, containing a triaxial accelerometer (as well as a gyroscope and magnetometer, but these data were not used in the current analysis) with a sampling rate of 30 Hz and a range of ±16*g* (*g* = 9.81 m/s^2^), and was worn on the nondominant wrist.

While wearing the devices, the participants completed VAS questionnaires, self-assessing their fatigue severity on a scale of 0 to 10, representing no fatigue to maximal imaginable fatigue. These fatigue-related PROs were self-reported up to 4 times a day: morning (6:00 am to 12:00 pm), afternoon (12:00 pm to 6:00 pm), evening (9:00 pm 12:00 am), and night (12:00 am to 6:00 am).

### Patient and public involvement

Members of the North East Sjogren’s Syndrome Association provided feedback on the study design. There was broad agreement and support of the study, with some minor suggestions which were implemented. Patients and/or the public were not involved in the conduct or reporting of this manuscript.

### Features

The HR, respiratory rate (RR), and activity type (ie, sitting, standing, and walking) were estimated by the VitalPatch sensor’s built-in software at sampling rates of 1 for every 4 seconds (0.25 Hz) for the HR and RR and 1 for every second (1 Hz) for the activity type. The instances across the study identified as sitting, standing, or walking, as a proportion of instances that were successfully identified, were estimated to give 3 activity-type measures for each participant. The mean, SD, and minimum and maximum HR and RR across the study were also calculated for each patient, giving 4 HR and 4 RR measures.

The triaxial accelerometer data from the wrist-worn ActiGraph were processed by the validated GGIR package (version 2.1) [28] to estimate the participants’ daily activity intensity [29,30] and sleep quality [31]. Ten activity intensity measures were extracted for each day, and then, the mean (weighted by wear-time) was found for each participant. These measures were the total duration of inactivity and light, moderate, and vigorous activity, as well as activity duration within several bout lengths: 1 minute to 10 minutes, 10 to 30 minutes, and ≥30 minutes for inactivity; 1 minute to 10 minutes and ≥10 minutes for light activity; and ≥1 minute for moderate-to-vigorous activity. The thresholds for the activity intensity levels were defined from the literature [[32], [33], [34]]. These activity intensity measures only included instances outside of the estimated sleep period time (SPT) window. Ten sleep measures were derived from the estimated SPT window; the time window starting at sleep onset and ending when waking up after the last sleep period of the night [31]. These were the time of sleep onset (start of the SPT), the time of wake up (end of the SPT), duration of the SPT in minutes, time spent asleep during the SPT in minutes, sleep efficiency (percentage of time asleep during the SPT), number of awakenings lasting at least 5 minutes, and time spent awake during the SPT, while inactive, in light activity, in moderate activity, and in vigorous activity in minutes. The activity intensity measures were weighted by the percentage of wear-time during the day and the sleep measures (excluding the sleep onset time, wake-up time, and sleep efficiency) were weighted by the percentage of wear-time during the SPT. The mean of these digital measures of activity and sleep were then calculated for each participant. This analysis of data from the 2 sensors gave a total of 31 digital measures per participant.

### Classification strategies

Two strategies were used to separate the participants: the persistent fatigue strategy and the overall fatigue strategy. For our proposed persistent fatigue strategy, the participants were assigned to persistent or nonpersistent fatigue groups. The participants were assigned to the persistent fatigue group if their minimum fatigue score was above a given threshold. Since there is no clear, valid threshold for fatigue VAS scores in the literature [35], our concept of persistent fatigue was tested with 4 different thresholds: 1, 2, 3, and 4. As seen in the Figure, this approach successfully grouped these participants into those with higher fatigue scores and less variation in their fatigue (shown by SD) and those with lower fatigue scores and more variation.

For the conventional, overall fatigue strategy, the participants were classed as having higher fatigue if their mean fatigue PRO score was above a given threshold. Again, 4 thresholds were tested: 4, 5, 6, and 7. These thresholds were selected to reflect the persistent fatigue strategy to give a relatively similar number of participants in each more vs less fatigue split.

### Statistical analysis

To analyse differences in estimated digital measures between fatigue groups, Welch *t* test and Mann-Whitney *U* tests were used. For each feature and threshold, the Shapiro-Wilk test was used to test both groups for normality. If both fatigue groups were normally distributed (Shapiro-Wilk test, *P* was <.05), Welch *t* test was used to compare the groups; otherwise, the Mann-Whitney *U* test was used. Since this was an exploratory study, multiple comparisons have not been accounted for. For clarity, the *P* values are reported, and a more stringent *P* value (.01) will be highlighted.

## RESULTS

### Clinical and demographic characteristics

Three of the participants were excluded due to having no fatigue VAS scores. These remaining 97 participants had a mean ± SD age of 59.7 ± 12.8 years and included 84 females and 12 males; 55 participants were not working, 22 worked part-time, and 19 worked full-time. One participant did not provide their demographic information.

Data were collected by the wrist-worn ActiGraph sensor for all 97 participants. However, there was substantial data loss for the chest-worn VitalPatch sensor due to technical challenges. This gave 72 participants with VitalPatch data, with a mean ± SD age of 59.9 ± 12.4 years and included 61 females and 10 males; 42 participants were not working, 18 worked part-time, and 11 worked full-time and included the participant without demographic information.

### PRO scores

Figure demonstrates the impact of the overall and persistent fatigue strategies on how the participants were grouped. The persistent strategy (Fig, C), allows the selection of persistently fatigued participants, whereas the overall strategy only selects the participants who, generally, reported higher levels of fatigue. Scatter plots of the remaining 6 thresholds tested in this analysis can be found in Supplementary Figures S1-S6, respectively.

**Figure:**
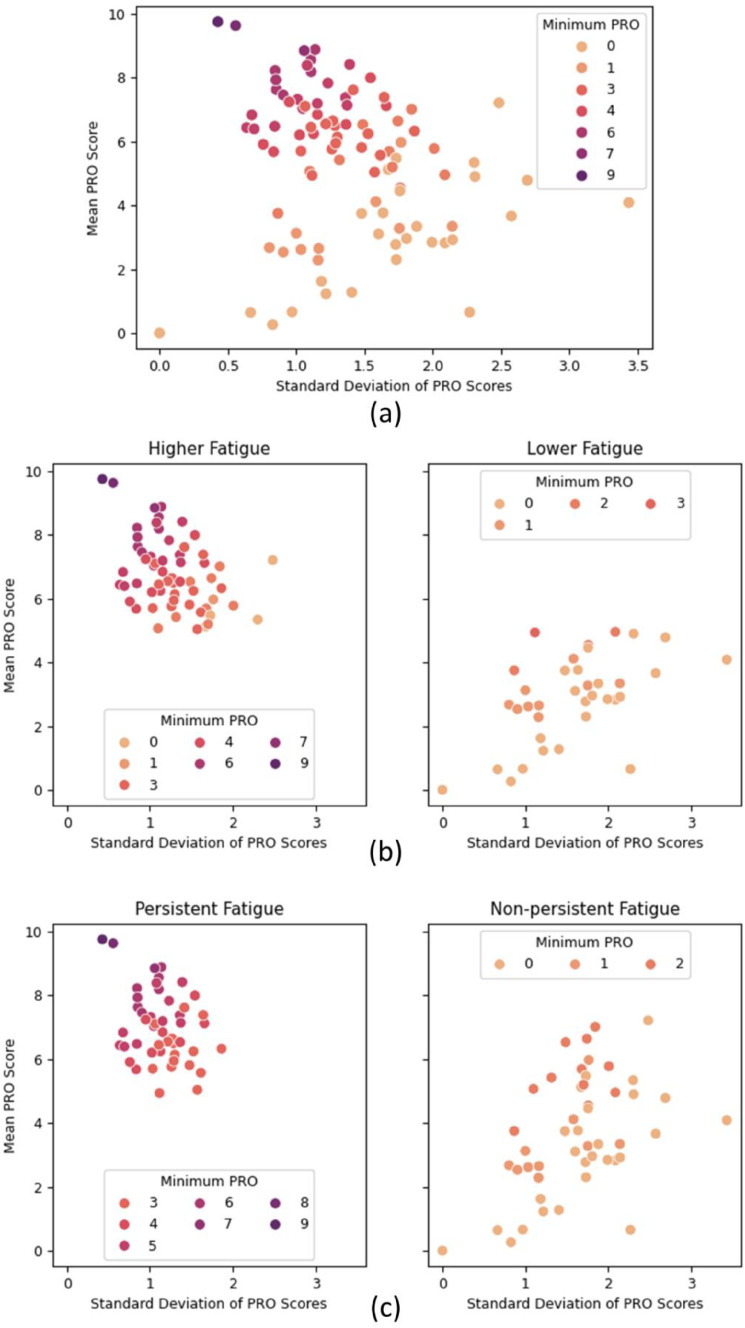
Scatter plots representing the participants’ fatigue PRO scores across the study. The participants’ mean fatigue score is plotted against the SD of the participants’ fatigue score, with the hue representing the participants’ minimum fatigue score. Each dot represents 1 participant. (A) All 97 participants. (B) An example of the overall strategy, with a threshold of 5. The participants in the higher fatigue class are shown on the left and the participants in the lower fatigue class on the right. (C) An example of the persistent strategy, with a threshold of 2. The participants grouped in the persistent fatigue class are shown on the left and the participants in the nonpersistent fatigue class on the right. PRO, patient-reported outcome.

The percentage of the 97 participants allocated into the persistent fatigue classes, as opposed to the nonpersistent fatigue class, by the persistent fatigue strategy, were threshold as follows: $(T_{P})=1$: 62%; $T_{P}=2$: 51%; $T_{P}=3$: 33%; and $T_{P}=4$: 23%. Similarly, the number of participants allocated into the higher fatigue class, as opposed to the lower fatigue class, by the overall fatigue strategy was follows: $T_{O}=4$: 71%; $T_{O}=5$: 63%; $T_{O}=6$: 44%; and $T_{O}=7$: 27%.

### Statistical analysis

From the 31 digital measures used in the current analysis, Table reports the *P* values and means for each measure that returned a statistically significant (*P* < .05) Welch *t* test or Mann-Whitney *U* test. Supplementary Table tabulates *P* values for all features and thresholds. The persistent fatigue strategy returned more statistically significant tests than the overall fatigue strategy, with 15 statistically significant (*P* < .05) tests from 10 measures compared with only 6 statistically significant tests from 5 measures. From the VitalPatch sensor, the only measures that were statistically significant (*P* < .05) were measures of HR (4 from the persistent strategy, 1 from the overall strategy), whereas the ActiGraph sensor returned significant measures from both the sleep (5 from the persistent strategy; 3 from the overall strategy) and activity intensity measures (4 from the persistent strategy; 2 from the overall strategy). Generally, mean HR was the most separable measure, with 3 thresholds from the persistent strategy and 1 threshold from the overall strategy returning tests with statistical significance.

**Table:** Summary of the statistically significant (P < .05) tests, comparing the descriptive statistic, the mean ± SD, or the median (IQR), and the P values

Persistence strategy: persistent vs nonpersistent fatigue						
Feature		*T_p_*	Statistic	Persistent	Nonpersistent	*P*
Activity intensity	Total duration inactive (min)	3	2.15^a^	572 ± 56^b^	543 ± 75^b^	.034
	Total duration light activity (min)	3	696^c^	208 ± 58^b^	235 (81)^d^	.008^e^
		4	−2.35^a^	212 ± 55^b^	245 ± 68^b^	.023
	Bouts ≥30 min inactive (min)	3^e^	2.19^a^	314 ± 132^b^	256 ± 106^b^	.033
	Bouts 1-10 min in light activity (min)	3^e^	664^c^	75 ± 30^b^	100 (42)^d^	.004^e^
		4	−2.34^a^	76 ± 29^b^	94 ± 5^b^	.024
HR	Mean (bpm)	2	2.21^a^	79.2 ± 9^b^	75.0 ± 7^b^	.031
		3^e^	2.75^a^	81.0 ± 8^b^	75.3 ± 8^b^	.009^e^
		4^e^	3.14^a^	82.3 ± 7^b^	75.7 ± 8^b^	.004^e^
	Minimum (bpm)	3	738^c^	54.0 ± 7^b^	52 (10)^d^	.035
Sleep	No. of awakenings	3	1297^c^	5.0 ± 2^b^	4.0 (2)^d^	.050
	Duration awake and inactive (min)	3	1313^c^	103 ± 35^b^	85 (37)^d^	.037
		4	1060^c^	105 ± 33^b^	86 (37)^d^	.043
	Length of sleep period (min)	3	2.15^a^	590 ± 41^b^	571 ± 40^b^	.036
	Sleep efficiency	4	588^c^	0.76 ± 0.07^b^	0.81 (0.09)^d^	.041

Overall strategy: higher vs lower fatigue						

Feature		*T_c_*	Statistic	Higher	Lower	*P*

Activity intensity	Bouts 10-30 min inactive (min)	5	829^c^	99 (35)^d^	109 ± 24^b^	.045
	Bouts 1-10 min in light activity (min)	5	835^c^	82 (52)^d^	97 ± 29^b^	.050
HR	Mean (bpm)	7	2.39^a^	80.6 ± 7^b^	75.9 ± 8^b^	.022
Sleep	Duration awake and in moderate activity (min)	5^e^	1457^c^	4.6 (4)^d^	2.9 (2)^d^	.007^e^
		6^e^	1558^c^	4.7 (5)^d^	3.0 (2)^d^	.004^e^
	Length of sleep period (min)	6	2.33^a^	588 ± 39^b^	569 ± 41^b^	.022

bpm, beats per minute; HR, heart rate.

a Welch *t* test was used.

b The parameter was normally distributed, and values are mean ± SD.

c Mann-Whitney *U* test.

d The parameter was not normally distributed, and values are median (IQR).

e *P* values <.01, and the *T_x_* column denotes the threshold.

A potential cofounder for activity and sleep is age. For both the overall and persistent fatigue strategies, the participants with more fatigue were significantly younger (*P* < .05) for all 4 thresholds. For example, for the overall fatigue threshold $T_{O}=5$, participants were aged 57.6 ± 12 vs 63.3 ± 13 years, and for the persistent threshold $T_{P}=2$, participants were aged 56.1 ± 13 vs 63.6 ± 11 years, more fatigue vs less fatigue, respectively. Therefore, the reduced activity intensity and sleep quality seen with persistent/higher fatigue groups could potentially be more substantial, since they are less active and have a poorer sleep quality despite being younger.

## DISCUSSION

This study explored the concept of persistent fatigue using objective measures of activity, sleep, and cardiorespiratory function from digital wearables in participants with SjD. In comparison with the conventional, overall fatigue strategy–higher vs lower fatigue–separating the participants based on the persistence of their fatigue revealed more statistically significant group differences with measures of activity, sleep, and cardiorespiratory function. This indicates that the isolating those who experience higher, less variable fatigue makes the digital measure’s group differences clearer, as opposed to simply isolating those with higher overall fatigue. Reviewing the literature, as discussed in the Introduction, showed that subjective measures of sleep and activity were inconsistent in reporting relationships with fatigue, whereas the objective digital measures found weak relationships, if any, between fatigue and measures of sleep and activity. The studies that used objective measures [12,[21], [22], [23]] analysed their data with correlations. Whereas the current study found that separating participants by their fatigue persistency revealed group differences with fatigue in SjD, potentially indicating that this approach is more robust to variations in sleep and activity than correlations.

When comparing the means of these features from the 2 fatigue classes in Table, in most cases, the trends from both strategies responded as anticipated based on understanding of fatigue in healthy individuals: those with more (persistent/higher) fatigue had reduced activity levels, a higher HR, and reduced sleep quality (more time spent in bed but less efficient, more time awake, and more movement during bedtime). The exception is the duration spent inactive for bouts 10 to 30 minutes for the overall threshold $T_{O}=5$, where those with higher fatigue spent less time inactive for these short breaks in activity. Considering those with higher overall fatigue spent more time in inactive in total (556 vs 547 minutes) and more time inactive in bouts ≥30 minutes (290 vs 249 minutes), it appears that those with higher overall fatigue engaged in longer bouts of inactivity rather than shorter bouts. When considering the activity intensity in the specific bout lengths for the persistent fatigue strategy, Table shows that those with persistent fatigue had a reduced duration in short (10-30 minutes) bouts of light activity and spent more time in longer (≥30 minutes) bouts of inactivity. Thus, those with persistent fatigue are possibly forgoing these short bouts of light activity, leading to the increased time in long bouts of inactivity, presenting a decrease in habitual activity with persistent fatigue that was not seen in overall fatigue.

Interestingly, Table shows group differences for the activity intensity captured by the ActiGraph, but these group differences were not reflected in the activity type captured by the VitalPatch. This is possibly due to the data loss with VitalPatch, or this change in activity is not represented by activity type. Take, for example, an individual who enjoys knitting while sitting and watching television, they may be less inclined to knit when they are experiencing more fatigue and will only be sat watching television. In this case, the VitalPatch would not register a change as they would be sat down either way, but the wrist-worn ActiGraph would record an increase in activity intensity when they are knitting. This example extends to many tasks, such as washing up, gardening, or ironing, where there may be a change in the amount of limbic movement without a change in posture, potentially leading to this incongruence in findings between the devices.

The current study found that activity intensity is reduced in those with persistent fatigue. In the literature, fatigue has been noted as a common barrier to physical activity [10]. Additionally, there is evidence that exercise may improve fatigue for some individuals with SjD [36,37], possibly due to an improvement of physical capacity, which has been shown to be related to the reduction of fatigue in SjD [38,39] and other rheumatologic disorders such as SLE [40,41]. Meanwhile, compared with self-assessed pain, mood, dryness, and brain fog, fatigue was found to have the largest impact on daily activities such as physical exercise, household chores, and hobbies [42]. Therefore, while there appears to be a relationship between activity and fatigue, it is not possible to identify the direction of causation in this relationship. It is possible, however, that the 2 influence each other, that is, increased fatigue leads to reduced activity as well as reduced activity leading to increased fatigue.

The current study found that those with increased fatigue had a poorer quality sleep, and this relationship was made clearer by separating the participants by the persistence of their fatigue. Two studies have been found to compare sleep measures from actigraphy and fatigue in SjD and found that wakings after sleep onset [21] and sleep efficiency [22] had no significant correlation with fatigue. This is congruent with the findings of the current study when the overall strategy (higher vs lower fatigue) was used, but when the persistent fatigue strategy was used sleep efficiency reported statistically significant between-group differences (*P* < .05). This finding is congruent with studies for other rheumatic diseases, where fragmented sleep has been found to have a positive correlation with fatigue [43] such as rheumatoid arthritis [44,45], fibromyalgia [46,47], and ankylosing spondylitis [48].

To our knowledge, this is the first study to explore the relationships between free-living HR and RR and patient-reported fatigue in SjD. However, the current study has shown that while RR indicated no relationship with fatigue, HR was higher in those with persistent fatigue. An increased HR correlating with increased fatigue was also reported in our previous work [24], indicating that the autonomic function has strong potential as a point of interest for future analysis. One measure that would be very interesting is heart rate variability (HRV) since our previous work reported that symptoms of autonomic dysfunction are correlated with fatigue in SjD [49]. There is also evidence that HRV is reduced with fatigue in multiple sclerosis [50] and chronic fatigue syndrome [51]. While the SD of the HR did not report any between-group differences in the current analysis, the minimum HR was higher for persistent fatigue strategy threshold $T_{P}=3$, therefore, more dedicated analysis of more complex measures of HRV may reveal a relationship.

Our findings show that a minimum VAS score of 3 was the most effective at identifying relationships with physiological parameters associated with fatigue, with 10 parameters relating to reduced limbic activity intensity, poorer sleep quality, and elevated HR showing statistical significance. Considering there is no established threshold for defining clinically meaningful fatigue from a VAS score, this threshold–or another measure of fatigue persistency–could be a suitable option. Further analysis should explore this approach as a possible threshold in a larger data set and consider the impact of reporting frequency, time of day, and study length.

An important limitation of the current study is the data loss experienced with the VitalPatch sensor, leading to 25 participants to be excluded from the activity type and cardiorespiratory function analysis. One should also consider that multiple comparisons have not been accounted for and the potential for multicollinearity in the statistically significant parameters reported in Table. For example, there is likely a relationship between number of awakenings, duration awake, and sleep efficiency. Another limitation is the mismatch of cofounders between the groups, such as age, sex, diagnosis duration, depression, and anxiety, which may have impacted the findings. These factors, especially the influences of depression and anxiety, should be accounted for and properly investigated in future, larger studies such as IDEA-FAST [52]. Another important focus of future work should be improving how participants are categorised into persistent vs nonpersistent fatigue groups. Using the minimum fatigue PRO score worked well for the participants in the current study, but this may not translate well to other sample populations. Possible solutions could be including PROs specific to the persistence of participants’ fatigue or using machine learning–based cluster analysis to group participants. Future research could also consider a 3-class problem: individuals with no fatigue, individuals with nonpersistent fatigue, and individuals with persistent fatigue. One of the most important lines of future research for the development of effectual interventions and early-warning predictions for patients, is identifying within-subject changes as opposed to the between-subject differences seen in the current analysis. This future line of work should also explore different approaches or time windows to aggregate the digital measures and fatigue scores. However, the current analysis has established that, despite challenges with PROs and inconsistency in the literature, digital wearables can identify fatigue from continuous measures of activity and physiology and are a promising tool in this important future line of study. Future research could also explore a single wrist sensor with a triaxial accelerometer and photoplethysmography, since this would capture all the key physiological parameters highlighted by this analysis: limbic activity intensity, sleep, and HR.

To conclude, separating participants with SjD based on the persistency of participant’s fatigue, rather than separating participants purely on their overall fatigue severity, revealed more between-group differences in real-world, objective measures of activity, sleep, and cardiorespiratory function. The persistent fatigue threshold that performed the best was $T_{P}=3$; therefore, it seems that a minimum VAS score of 3 (for a study lasting 2 weeks) could be a clinically relevant threshold for fatigue research. The trends were, for the most part, as expected; increased fatigue was seen with reduced activity and sleep quality and an increased HR. The current analysis has identified HRV as an interesting measure for future analysis, as well as the need of an improved definition of persistent fatigue going forward, and an exploration of within-subject changes in fatigue. Overall, the current analysis has presented an alternative approach to overcome the challenges of conducting research with subjective measures of fatigue; leading to an objective, continuous assessment of fatigue using digital wearables.

## CRediT authorship contribution statement

**Chloe Hinchliffe:** Writing – review & editing, Writing – original draft, Visualization, Methodology, Investigation, Formal analysis. **Bing Zhai:** Writing – review & editing, Formal analysis, Data curation. **Victoria Macrae:** Data curation. **Jade Walton:** Data curation. **Silvia Del Din:** Writing – review & editing, Supervision, Conceptualization. **Wan-Fai Ng:** Writing – review & editing, Supervision, Funding acquisition, Data curation, Conceptualization.

## Appendix Supplementary materials

Image, application 1

## Footnotes

Supplementary material associated with this article can be found in the online version at doi:10.1016/j.ero.2025.11.004.
