# Supplementary material for: Can real-world measures of activity, sleep, and cardiorespiratory function stratify Sjogren’s disease participants with persistent fatigue? Insights from the BRC tools study
Source: EULAR Rheumatol Open. 2025 Nov 30;1(4):422–9. doi: 10.1016/j.ero.2025.11.004 (PMC13292492; doi:10.1016/j.ero.2025.11.004)
Supplement: Supplementary file 1 [file mmc1.docx]

Can Real-World Measures of Activity, Sleep, and Cardiorespiratory Function Stratify Sjogren’s Disease Participants with Persistent Fatigue? Insights from the BRC Tools Study:

**Supplementary Materials**

Inclusion criteria:

1. Age ≥ 18 years.

2. Fulfils the American European Consensus Group (AECG) classification criteria for primary Sjogren’s disease.

3. Willing and able to provide informed written consent and attend the clinic for visits.

Exclusion criteria:

1. Hospital Anxiety and Depression Score (HADS≥11).

2. Fibromyalgia or chronic pain syndromes.

3. History of cerebrovascular diseases, dementia (mini mental state examination score ≤ 24), other central nervous system injuries/disorders that impair neurocognitive function.

4. A history of severe skin allergies.

Rationale behind the exclusion criteria. Depression, anxiety and chronic pain syndrome are important confounders of fatigue. Significant cerebral diseases will affect the performance of neuropsychological tests. There is a potential that a skin reaction may occur when in contact with the VitalPatch; patients with known severe skin allergies are excluded.


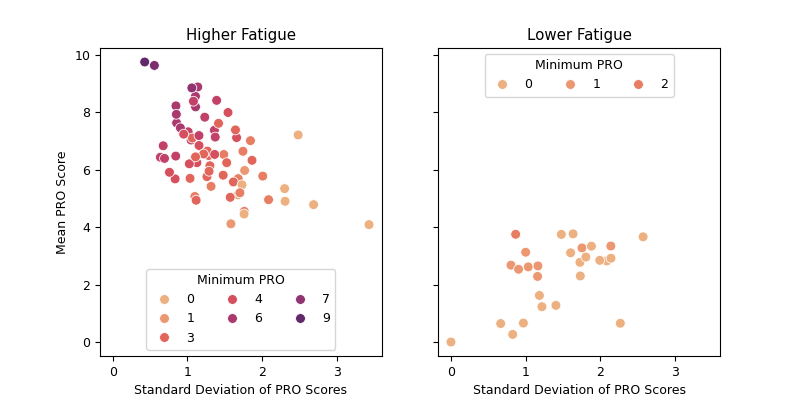


S1 Scatter plots representing the participants’ fatigue PRO scores across the study, for the **overall strategy**, with a **threshold of 4**. The participants’ mean fatigue score is plotted against the SD of the participants’ fatigue score, with the hue representing the participants’ minimum fatigue score. Each dot represents one participant. The participants in the higher fatigue class are shown on the left and the participants are grouped in the lower fatigue class on the right.


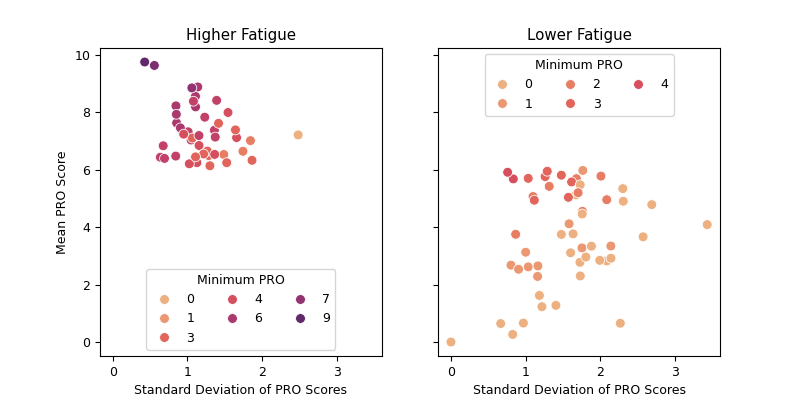


S2 Scatter plots representing the participants’ fatigue PRO scores across the study, for the **overall strategy**, with a **threshold of 6**. The participants’ mean fatigue score is plotted against the SD of the participants’ fatigue score, with the hue representing the participants’ minimum fatigue score. Each dot represents one participant. The participants in the higher fatigue class are shown on the left and the participants are grouped in the lower fatigue class on the right.


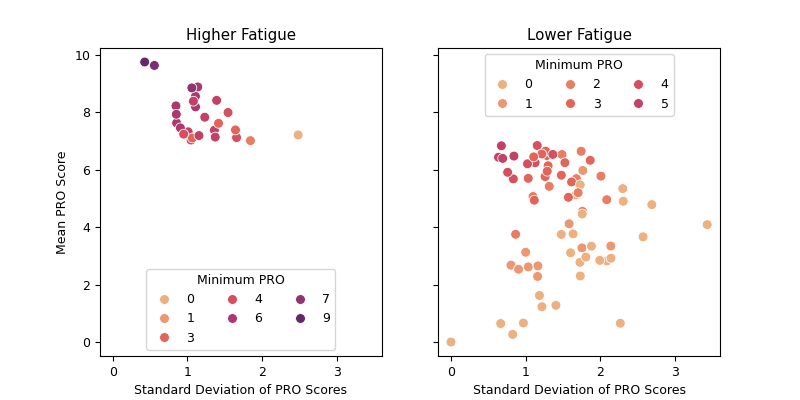


S3 Scatter plots representing the participants’ fatigue PRO scores across the study, for the **overall strategy**, with a **threshold of 7**. The participants’ mean fatigue score is plotted against the SD of the participants’ fatigue score, with the hue representing the participants’ minimum fatigue score. Each dot represents one participant. The participants in the higher fatigue class are shown on the left and the participants are grouped in the lower fatigue class on the right.


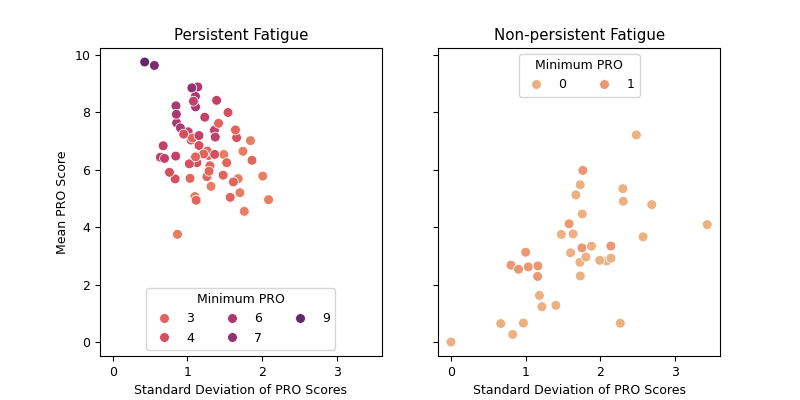


S4 Scatter plots representing the participants’ fatigue PRO scores across the study, for the **persistent strategy**, with a **threshold of 1**. The participants’ mean fatigue score is plotted against the SD of the participants’ fatigue score, with the hue representing the participants’ minimum fatigue score. Each dot represents one participant. The participants in the persistent fatigue class are shown on the left and the participants are grouped in the non-persistent fatigue class on the right.


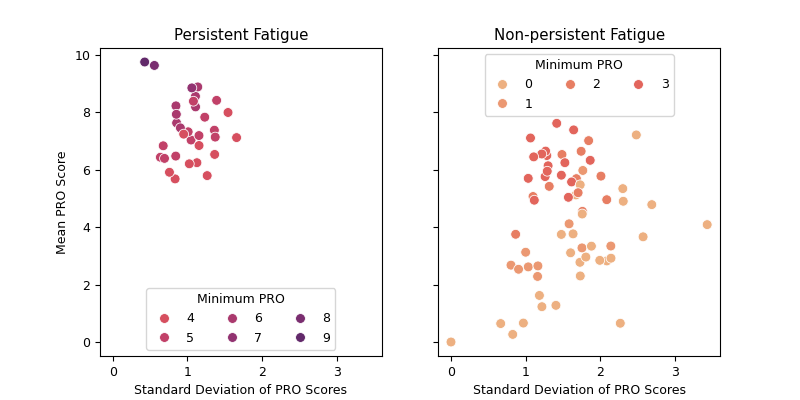


S5 Scatter plots representing the participants’ fatigue PRO scores across the study, for the **persistent strategy**, with a **threshold of 3**. The participants’ mean fatigue score is plotted against the SD of the participants’ fatigue score, with the hue representing the participants’ minimum fatigue score. Each dot represents one participant. The participants in the persistent fatigue class are shown on the left and the participants are grouped in the non-persistent fatigue class on the right.


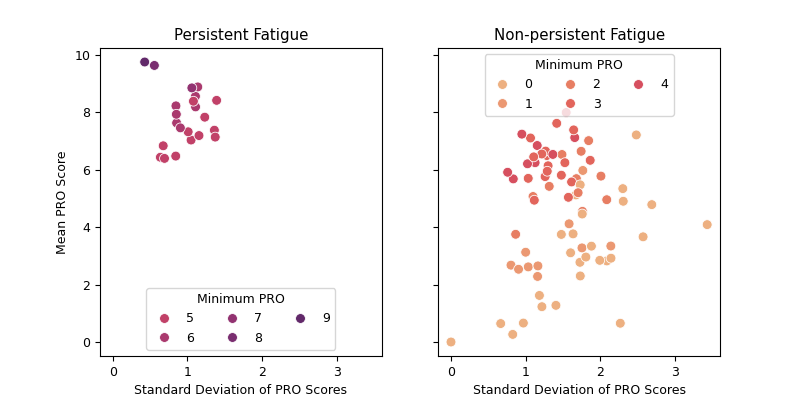


S6 Scatter plots representing the participants’ fatigue PRO scores across the study, for the **persistent strategy**, with a **threshold of 4**. The participants’ mean fatigue score is plotted against the SD of the participants’ fatigue score, with the hue representing the participants’ minimum fatigue score. Each dot represents one participant. The participants in the persistent fatigue class are shown on the left and the participants are grouped in the non-persistent fatigue class on the right.

Supplementary Table Results of the statistical tests. Reporting the descriptive statistic, the mean ± SD (if normally distributed) or the median ± interquartile range (IQR) (if not normally distributed), and the p-values. The $T_{X}$ column denotes the threshold _T denotes normal distribution and _F denotes not normal distribution, if both columns report _T then Welch’s t-test was used otherwise Mann-Whitney U test was used.

| **Persistence Strategy: Persistent vs. Non-persistent Fatigue** | | | | | | |
| --- | --- | --- | --- | --- | --- | --- |
| **Feature** | | **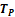** | **Statistic** | **Persistent** | **Non-persistent** | **p-value** |
| Activity_intensity | dur_day_MVPA_bts_1_min | 1 | 1026 | 23.87+-21.4_F | 18.6+-21.5_F | 0.5352 |
|  | dur_day_IN_bts_30_min | 1 | 1.94 | 248.0+-91.7_T | 291.43+-128.8_T | 0.056 |
|  | dur_day_IN_bts_10_30_min | 1 | 963 | 105.72+-23.3_T | 101.89+-33.9_F | 0.2766 |
|  | dur_day_IN_bts_1_10_min | 1 | -1.87 | 122.51+-26.6_T | 110.73+-35.1_T | 0.0644 |
|  | dur_day_LIG_bts_10_min | 1 | 1079 | 19.19+-29.5_F | 18.67+-27.6_F | 0.8208 |
|  | dur_day_LIG_bts_1_10_min | 1 | 872 | 96.51+-30.7_T | 85.07+-51.1_F | 0.0778 |
|  | dur_day_total_IN_min | 1 | 0.98 | 543.84+-69.4_T | 558.2+-71.4_T | 0.3307 |
|  | dur_day_total_LIG_min | 1 | 879 | 251.44+-56.3_T | 232.34+-90.0_F | 0.0869 |
|  | dur_day_total_MOD_min | 1 | -0.73 | 63.61+-29.4_T | 59.14+-29.6_T | 0.4705 |
|  | dur_day_total_VIG_min | 1 | 1101.5 | 0.51+-1.2_F | 0.34+-1.1_F | 0.9526 |
| Activity_type | time_sed | 1 | 692 | 0.44+-0.1_F | 0.48+-0.1_T | 0.273 |
|  | time_stand | 1 | 520 | 0.52+-0.2_F | 0.49+-0.1_T | 0.3636 |
|  | time_walk | 1 | 525 | 0.02+-0.0_F | 0.02+-0.0_F | 0.3953 |
| HR | mean | 1 | 0.84 | 76.09+-7.5_T | 77.73+-8.7_T | 0.4018 |
|  | sd | 1 | 609 | 12.71+-3.1_T | 12.51+-4.7_F | 0.902 |
|  | min | 1 | 724 | 49.42+-9.1_T | 54.0+-8.8_F | 0.1404 |
|  | max | 1 | 609.5 | 161.12+-26.3_T | 163.5+-50.0_F | 0.8971 |
| Resp | mean | 1 | 637 | 17.33+-3.0_F | 17.41+-1.4_T | 0.6517 |
|  | sd | 1 | 460 | 3.63+-0.8_F | 3.48+-0.4_T | 0.107 |
|  | min | 1 | 616.5 | 4.0+-1.0_F | 4.0+-2.0_F | 0.8096 |
|  | max | 1 | 478 | 37.15+-3.1_T | 35.0+-5.8_F | 0.1586 |
| Sleep | sleeponset | 1 | 1012 | 22.51+-0.5_F | 22.42+-0.4_F | 0.469 |
|  | wakeup | 1 | 1.29 | 32.22+-0.5_T | 32.36+-0.6_T | 0.2022 |
|  | sleep_efficiency | 1 | 924 | 0.81+-0.1_F | 0.78+-0.1_T | 0.1683 |
|  | N_atleast5minwakenight | 1 | 1137.5 | 4.08+-2.3_F | 4.45+-1.9_T | 0.8411 |
|  | dur_spt_sleep_min | 1 | -0.19 | 452.95+-42.3_T | 451.25+-46.0_T | 0.8531 |
|  | dur_spt_wake_IN_min | 1 | 1243 | 88.17+-26.6_T | 91.95+-44.3_F | 0.3251 |
|  | dur_spt_wake_LIG_min | 1 | 1244 | 21.74+-15.8_F | 25.73+-15.7_F | 0.3215 |
|  | dur_spt_wake_MOD_min | 1 | 1351 | 3.02+-2.1_F | 4.34+-3.3_F | 0.0741 |
|  | dur_spt_wake_VIG_min | 1 | 1208.5 | 0.01+-0.0_F | 0.02+-0.1_F | 0.4612 |
|  | dur_spt_min | 1 | 1.04 | 572.0+-37.2_T | 580.61+-43.3_T | 0.3023 |
| Activity_intensity | dur_day_MVPA_bts_1_min | 2 | 1077 | 23.43+-20.4_F | 18.57+-22.4_F | 0.4773 |
|  | dur_day_IN_bts_30_min | 2 | 1.25 | 259.92+-92.7_T | 289.51+-136.9_T | 0.2151 |
|  | dur_day_IN_bts_10_30_min | 2 | 1076 | 104.16+-24.3_T | 103.57+-34.9_F | 0.4728 |
|  | dur_day_IN_bts_1_10_min | 2 | -0.99 | 118.5+-27.6_T | 112.02+-36.7_T | 0.3269 |
|  | dur_day_LIG_bts_10_min | 2 | 1152 | 18.33+-33.0_F | 19.33+-23.5_F | 0.8654 |
|  | dur_day_LIG_bts_1_10_min | 2 | 906 | 95.55+-28.7_T | 82.21+-52.5_F | 0.0518 |
|  | dur_day_total_IN_min | 2 | 0.83 | 546.7+-68.2_T | 558.62+-73.1_T | 0.4085 |
|  | dur_day_total_LIG_min | 2 | 925 | 249.01+-54.6_T | 233.26+-90.3_F | 0.0707 |
|  | dur_day_total_MOD_min | 2 | -0.84 | 63.39+-28.1_T | 58.35+-30.9_T | 0.4024 |
|  | dur_day_total_VIG_min | 2 | 1228.5 | 0.51+-1.2_F | 0.35+-1.1_F | 0.7075 |
| Activity_type | time_sed | 2 | 723 | 0.44+-0.2_F | 0.48+-0.1_T | 0.4014 |
|  | time_stand | 2 | 595 | 0.52+-0.2_F | 0.49+-0.1_T | 0.5543 |
|  | time_walk | 2 | 579 | 0.02+-0.0_F | 0.02+-0.0_F | 0.4404 |
| HR | mean | 2 | 2.21 | 75.04+-7.3_T | 79.24+-8.8_T | 0.0306 |
|  | sd | 2 | 1.15 | 12.41+-3.0_T | 13.41+-4.2_T | 0.2544 |
|  | min | 2 | 755.5 | 50.33+-8.5_T | 55.0+-9.2_F | 0.2273 |
|  | max | 2 | 695.5 | 159.0+-46.5_F | 163.5+-46.5_F | 0.5954 |
| Resp | mean | 2 | 722 | 17.33+-2.9_F | 17.51+-1.3_T | 0.4078 |
|  | sd | 2 | 574 | 3.62+-0.7_F | 3.5+-0.4_T | 0.4078 |
|  | min | 2 | 642.5 | 4.0+-1.0_F | 4.0+-2.0_F | 0.9487 |
|  | max | 2 | 643.5 | 36.47+-3.3_T | 36.0+-6.5_F | 0.9638 |
| Sleep | sleeponset | 2 | 1044 | 22.51+-0.5_F | 22.43+-0.4_F | 0.3427 |
|  | wakeup | 2 | 1.54 | 32.22+-0.5_T | 32.39+-0.6_T | 0.1282 |
|  | sleep_efficiency | 2 | -1.27 | 0.79+-0.1_T | 0.78+-0.1_T | 0.2077 |
|  | N_atleast5minwakenight | 2 | 1234.5 | 4.03+-2.0_F | 4.53+-2.0_T | 0.6756 |
|  | dur_spt_sleep_min | 2 | -0.03 | 452.02+-42.4_T | 451.78+-46.7_T | 0.9784 |
|  | dur_spt_wake_IN_min | 2 | 1339 | 88.25+-25.0_T | 97.82+-49.5_F | 0.241 |
|  | dur_spt_wake_LIG_min | 2 | 1250 | 23.53+-15.5_F | 25.17+-16.7_F | 0.5959 |
|  | dur_spt_wake_MOD_min | 2 | 1364 | 3.08+-2.3_F | 4.12+-3.1_F | 0.1761 |
|  | dur_spt_wake_VIG_min | 2 | 1334 | 0.01+-0.0_F | 0.03+-0.1_F | 0.2499 |
|  | dur_spt_min | 2 | 1.26 | 572.02+-37.0_T | 582.52+-44.5_T | 0.2091 |
| Activity_intensity | dur_day_MVPA_bts_1_min | 3 | 826 | 23.87+-21.6_F | 12.83+-18.9_F | 0.1014 |
|  | dur_day_IN_bts_30_min | 3 | 2.19 | 255.53+-105.5_T | 314.13+-131.7_T | 0.0328 |
|  | dur_day_IN_bts_10_30_min | 3 | 957 | 104.09+-26.7_T | 103.63+-37.0_F | 0.5267 |
|  | dur_day_IN_bts_1_10_min | 3 | -1.2 | 118.42+-26.8_T | 108.73+-41.5_T | 0.2354 |
|  | dur_day_LIG_bts_10_min | 3 | 889 | 20.45+-38.1_F | 17.84+-17.1_F | 0.2482 |
|  | dur_day_LIG_bts_1_10_min | 3 | 664 | 100.33+-42.1_F | 74.54+-29.8_T | 0.004 |
|  | dur_day_total_IN_min | 3 | 2.15 | 543.05+-75.3_T | 572.35+-56.0_T | 0.0344 |
|  | dur_day_total_LIG_min | 3 | 696 | 235.54+-81.6_F | 207.58+-57.6_T | 0.0084 |
|  | dur_day_total_MOD_min | 3 | 816 | 62.15+-45.3_F | 52.56+-26.9_T | 0.0864 |
|  | dur_day_total_VIG_min | 3 | 960.5 | 0.52+-1.2_F | 0.3+-0.9_F | 0.5444 |
| Activity_type | time_sed | 3 | 560 | 0.45+-0.2_F | 0.45+-0.1_T | 0.9711 |
|  | time_stand | 3 | 1.07 | 0.49+-0.2_T | 0.52+-0.1_T | 0.289 |
|  | time_walk | 3 | 421 | 0.03+-0.0_F | 0.02+-0.0_T | 0.0864 |
| HR | mean | 3 | 2.75 | 75.32+-7.6_T | 81.01+-8.4_T | 0.0089 |
|  | sd | 3 | 578 | 12.33+-4.2_F | 12.94+-3.9_T | 0.8657 |
|  | min | 3 | 738 | 52.0+-10.0_F | 54.04+-6.7_T | 0.0352 |
|  | max | 3 | 572.5 | 163.0+-56.0_F | 162.83+-23.4_T | 0.918 |
| Resp | mean | 3 | 552 | 17.43+-2.6_F | 17.33+-1.4_T | 0.8943 |
|  | sd | 3 | 532 | 3.56+-0.7_F | 3.5+-0.3_T | 0.7081 |
|  | min | 3 | 579 | 4.0+-1.0_F | 4.0+-1.5_F | 0.8362 |
|  | max | 3 | 571 | 36.0+-4.0_F | 36.61+-3.6_T | 0.9322 |
| Sleep | sleeponset | 3 | 818 | 22.51+-0.5_F | 22.36+-0.3_T | 0.0892 |
|  | wakeup | 3 | 1.96 | 32.22+-0.5_T | 32.47+-0.6_T | 0.0556 |
|  | sleep_efficiency | 3 | 800.5 | 0.81+-0.1_F | 0.77+-0.1_T | 0.0667 |
|  | N_atleast5minwakenight | 3 | 1296.5 | 3.98+-2.2_F | 5.02+-2.1_T | 0.0495 |
|  | dur_spt_sleep_min | 3 | 0.28 | 450.97+-42.4_T | 453.79+-48.8_T | 0.7808 |
|  | dur_spt_wake_IN_min | 3 | 1313 | 84.76+-36.7_F | 103.02+-34.7_T | 0.0365 |
|  | dur_spt_wake_LIG_min | 3 | 1141 | 24.56+-14.5_F | 27.86+-12.1_T | 0.4406 |
|  | dur_spt_wake_MOD_min | 3 | 1178 | 3.18+-2.8_F | 4.04+-3.1_F | 0.2914 |
|  | dur_spt_wake_VIG_min | 3 | 1152 | 0.02+-0.1_F | 0.02+-0.1_F | 0.3864 |
|  | dur_spt_min | 3 | 2.15 | 571.1+-40.0_T | 589.97+-40.9_T | 0.0356 |
| Activity_intensity | dur_day_MVPA_bts_1_min | 4 | 676 | 23.07+-21.6_F | 12.83+-18.8_F | 0.2008 |
|  | dur_day_IN_bts_30_min | 4 | 1.59 | 264.85+-117.4_T | 309.02+-113.6_T | 0.1204 |
|  | dur_day_IN_bts_10_30_min | 4 | 745 | 101.8+-33.4_F | 98.27+-23.7_T | 0.4934 |
|  | dur_day_IN_bts_1_10_min | 4 | -0.68 | 116.66+-29.9_T | 110.34+-40.5_T | 0.5021 |
|  | dur_day_LIG_bts_10_min | 4 | 682 | 19.94+-29.8_F | 16.33+-16.0_F | 0.2196 |
|  | dur_day_LIG_bts_1_10_min | 4 | -2.34 | 94.0+-35.9_T | 76.4+-29.5_T | 0.0243 |
|  | dur_day_total_IN_min | 4 | 1.74 | 547.35+-75.2_T | 571.02+-49.1_T | 0.0874 |
|  | dur_day_total_LIG_min | 4 | -2.35 | 245.09+-68.3_T | 211.77+-55.1_T | 0.0234 |
|  | dur_day_total_MOD_min | 4 | -1.87 | 63.56+-30.2_T | 51.59+-25.2_T | 0.0686 |
|  | dur_day_total_VIG_min | 4 | 773.5 | 0.51+-1.2_F | 0.31+-0.8_F | 0.6604 |
| Activity_type | time_sed | 4 | 385 | 0.46+-0.2_F | 0.43+-0.1_T | 0.3972 |
|  | time_stand | 4 | 1.9 | 0.49+-0.2_T | 0.55+-0.1_T | 0.0655 |
|  | time_walk | 4 | 337 | 0.03+-0.0_F | 0.02+-0.0_T | 0.1345 |
| HR | mean | 4 | 3.14 | 75.66+-8.0_T | 82.31+-7.3_T | 0.0041 |
|  | sd | 4 | 524 | 12.33+-4.2_F | 13.76+-3.9_T | 0.3065 |
|  | min | 4 | 583.5 | 52.0+-10.0_F | 57.0+-8.0_F | 0.0669 |
|  | max | 4 | 481.5 | 161.5+-49.2_F | 165.19+-23.8_T | 0.6538 |
| Resp | mean | 4 | 399 | 17.44+-2.4_F | 17.14+-1.5_T | 0.5112 |
|  | sd | 4 | 418 | 3.58+-0.6_F | 3.5+-0.3_T | 0.6895 |
|  | min | 4 | 498.5 | 4.0+-1.0_F | 4.5+-2.0_F | 0.4395 |
|  | max | 4 | 424 | 36.0+-5.2_F | 36.19+-3.4_T | 0.7488 |
| Sleep | sleeponset | 4 | 741 | 22.45+-0.5_F | 22.38+-0.3_T | 0.4719 |
|  | wakeup | 4 | 0.9 | 32.27+-0.5_T | 32.42+-0.7_T | 0.3739 |
|  | sleep_efficiency | 4 | 587.5 | 0.81+-0.1_F | 0.76+-0.1_T | 0.0412 |
|  | N_atleast5minwakenight | 4 | 1.67 | 4.23+-1.8_T | 5.09+-2.2_T | 0.1063 |
|  | dur_spt_sleep_min | 4 | -0.27 | 452.63+-42.7_T | 449.41+-50.8_T | 0.7888 |
|  | dur_spt_wake_IN_min | 4 | 1060 | 85.55+-37.2_F | 104.57+-33.1_T | 0.0434 |
|  | dur_spt_wake_LIG_min | 4 | 1038 | 22.49+-14.5_F | 30.69+-12.8_T | 0.0672 |
|  | dur_spt_wake_MOD_min | 4 | 984 | 3.2+-2.8_F | 4.38+-3.3_F | 0.1721 |
|  | dur_spt_wake_VIG_min | 4 | 871 | 0.02+-0.1_F | 0.02+-0.1_F | 0.6915 |
|  | dur_spt_min | 4 | 1.73 | 573.55+-41.1_T | 590.19+-39.3_T | 0.0926 |
| **Overall Strategy: Higher vs. Lower Fatigue** | | | | | | |
| **Feature** | | **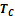** | **Statistic** | **Higher** | **Lower** | **p-value** |
| Activity_intensity | dur_day_MVPA_bts_1_min | 4 | 923 | 22.2+-15.1_T | 18.57+-23.4_F | 0.7351 |
|  | dur_day_IN_bts_30_min | 4 | 1.35 | 251.69+-100.8_T | 284.27+-123.0_T | 0.1819 |
|  | dur_day_IN_bts_10_30_min | 4 | 804 | 107.19+-24.5_T | 101.31+-32.9_F | 0.1985 |
|  | dur_day_IN_bts_1_10_min | 4 | -1.42 | 122.13+-29.0_T | 112.42+-33.6_T | 0.1596 |
|  | dur_day_LIG_bts_10_min | 4 | 984 | 15.76+-34.7_F | 19.33+-25.4_F | 0.8892 |
|  | dur_day_LIG_bts_1_10_min | 4 | 764 | 105.35+-60.0_F | 85.14+-49.8_F | 0.1087 |
|  | dur_day_total_IN_min | 4 | 0.36 | 548.52+-74.9_T | 554.42+-69.3_T | 0.7208 |
|  | dur_day_total_LIG_min | 4 | 777 | 251.17+-56.8_T | 232.55+-78.4_F | 0.1334 |
|  | dur_day_total_MOD_min | 4 | 938 | 60.92+-26.1_T | 57.89+-44.2_F | 0.8267 |
|  | dur_day_total_VIG_min | 4 | 852.5 | 0.67+-1.3_F | 0.33+-1.1_F | 0.3683 |
| Activity_type | time_sed | 4 | 1.21 | 0.44+-0.2_T | 0.49+-0.1_T | 0.2369 |
|  | time_stand | 4 | -1.11 | 0.53+-0.1_T | 0.49+-0.1_T | 0.2755 |
|  | time_walk | 4 | 434 | 0.03+-0.0_F | 0.02+-0.0_F | 0.2824 |
| HR | mean | 4 | 1.41 | 74.98+-8.0_T | 77.97+-8.3_T | 0.1686 |
|  | sd | 4 | 532 | 12.7+-3.2_T | 12.76+-4.8_F | 0.885 |
|  | min | 4 | 648.5 | 48.75+-9.6_T | 55.0+-9.0_F | 0.1069 |
|  | max | 4 | 583 | 157.6+-26.6_T | 164.5+-49.5_F | 0.4305 |
| Resp | mean | 4 | 601 | 16.57+-3.0_F | 17.47+-1.4_T | 0.3115 |
|  | sd | 4 | 367 | 3.63+-0.7_F | 3.41+-0.6_F | 0.0552 |
|  | min | 4 | 478 | 4.0+-1.2_F | 4.0+-1.2_F | 0.5515 |
|  | max | 4 | 499 | 36.55+-3.6_T | 36.0+-5.2_F | 0.7954 |
| Sleep | sleeponset | 4 | 904 | 22.51+-0.5_F | 22.43+-0.4_F | 0.6244 |
|  | wakeup | 4 | 0.88 | 32.23+-0.5_T | 32.34+-0.6_T | 0.3854 |
|  | sleep_efficiency | 4 | -0.78 | 0.79+-0.1_T | 0.78+-0.1_T | 0.4403 |
|  | N_atleast5minwakenight | 4 | 1010 | 3.98+-1.3_F | 4.46+-1.9_T | 0.7291 |
|  | dur_spt_sleep_min | 4 | 0.1 | 451.15+-44.9_T | 452.2+-44.5_T | 0.917 |
|  | dur_spt_wake_IN_min | 4 | 1056 | 88.84+-27.7_T | 92.08+-43.7_F | 0.4761 |
|  | dur_spt_wake_LIG_min | 4 | 1093 | 21.16+-15.3_F | 26.29+-13.9_F | 0.3139 |
|  | dur_spt_wake_MOD_min | 4 | 1173 | 2.97+-1.9_F | 3.95+-3.1_F | 0.1002 |
|  | dur_spt_wake_VIG_min | 4 | 1015.5 | 0.02+-0.0_F | 0.02+-0.1_F | 0.6929 |
|  | dur_spt_min | 4 | 0.93 | 571.35+-39.8_T | 579.75+-41.7_T | 0.3572 |
| Activity_intensity | dur_day_MVPA_bts_1_min | 5 | 1075 | 23.43+-20.1_F | 18.64+-23.4_F | 0.8666 |
|  | dur_day_IN_bts_30_min | 5 | 1.81 | 249.33+-91.7_T | 289.93+-128.6_T | 0.074 |
|  | dur_day_IN_bts_10_30_min | 5 | 829 | 109.18+-24.4_T | 99.35+-34.9_F | 0.045 |
|  | dur_day_IN_bts_1_10_min | 5 | -1.6 | 121.6+-27.0_T | 111.46+-35.0_T | 0.1136 |
|  | dur_day_LIG_bts_10_min | 5 | 1033 | 19.82+-29.3_F | 18.02+-29.3_F | 0.6301 |
|  | dur_day_LIG_bts_1_10_min | 5 | 835 | 97.07+-28.6_T | 82.21+-51.8_F | 0.05 |
|  | dur_day_total_IN_min | 5 | 0.64 | 546.82+-68.2_T | 556.2+-72.4_T | 0.524 |
|  | dur_day_total_LIG_min | 5 | 842 | 251.27+-52.8_T | 231.42+-88.1_F | 0.0564 |
|  | dur_day_total_MOD_min | 5 | -0.01 | 60.87+-27.6_T | 60.83+-30.7_T | 0.9948 |
|  | dur_day_total_VIG_min | 5 | 1102 | 0.51+-1.2_F | 0.35+-1.1_F | 0.9791 |
| Activity_type | time_sed | 5 | -0.48 | 0.48+-0.2_T | 0.47+-0.1_T | 0.6372 |
|  | time_stand | 5 | 0.64 | 0.48+-0.2_T | 0.51+-0.1_T | 0.5264 |
|  | time_walk | 5 | 457 | 0.03+-0.0_F | 0.02+-0.0_F | 0.0995 |
| HR | mean | 5 | 1.4 | 75.36+-7.9_T | 78.15+-8.4_T | 0.1659 |
|  | sd | 5 | 0.73 | 12.51+-3.1_T | 13.13+-4.0_T | 0.4672 |
|  | min | 5 | 696 | 52.5+-9.8_F | 52.24+-7.4_T | 0.2521 |
|  | max | 5 | 721 | 155.35+-27.9_T | 165.5+-50.0_F | 0.1496 |
| Resp | mean | 5 | 626 | 17.55+-3.2_F | 17.4+-1.3_T | 0.7471 |
|  | sd | 5 | 469 | 3.62+-0.8_F | 3.41+-0.6_F | 0.1319 |
|  | min | 5 | 503 | 4.0+-2.0_F | 4.0+-1.0_F | 0.206 |
|  | max | 5 | 562 | 36.65+-3.5_T | 36.0+-5.0_F | 0.6754 |
| Sleep | sleeponset | 5 | 936 | 22.52+-0.3_F | 22.42+-0.4_F | 0.2278 |
|  | wakeup | 5 | 1.18 | 32.22+-0.5_T | 32.36+-0.6_T | 0.2402 |
|  | sleep_efficiency | 5 | 888 | 0.82+-0.1_F | 0.78+-0.1_T | 0.1177 |
|  | N_atleast5minwakenight | 5 | 1121 | 4.1+-1.6_F | 4.47+-2.0_T | 0.8666 |
|  | dur_spt_sleep_min | 5 | -0.32 | 453.74+-43.6_T | 450.81+-45.2_T | 0.7532 |
|  | dur_spt_wake_IN_min | 5 | 1261 | 87.31+-25.6_T | 95.18+-47.5_F | 0.225 |
|  | dur_spt_wake_LIG_min | 5 | 1240 | 21.72+-15.8_F | 26.29+-13.7_F | 0.2907 |
|  | dur_spt_wake_MOD_min | 5 | 1457 | 2.85+-1.5_F | 4.6+-3.7_F | 0.0074 |
|  | dur_spt_wake_VIG_min | 5 | 1296 | 0.01+-0.0_F | 0.02+-0.1_F | 0.1354 |
|  | dur_spt_min | 5 | 1.08 | 571.66+-37.6_T | 580.67+-43.0_T | 0.2833 |
| Activity_intensity | dur_day_MVPA_bts_1_min | 6 | 1137 | 20.82+-19.7_F | 19.3+-27.3_F | 0.8645 |
|  | dur_day_IN_bts_30_min | 6 | 0.97 | 264.32+-108.2_T | 288.1+-128.2_T | 0.3341 |
|  | dur_day_IN_bts_10_30_min | 6 | 898 | 106.59+-23.2_T | 93.25+-38.4_F | 0.0566 |
|  | dur_day_IN_bts_1_10_min | 6 | -1.27 | 119.07+-28.2_T | 110.39+-37.0_T | 0.2069 |
|  | dur_day_LIG_bts_10_min | 6 | 1105 | 17.56+-30.3_F | 19.33+-25.4_F | 0.6869 |
|  | dur_day_LIG_bts_1_10_min | 6 | 893 | 95.55+-33.1_T | 84.99+-53.0_F | 0.0521 |
|  | dur_day_total_IN_min | 6 | -0.01 | 552.76+-71.4_T | 552.66+-70.5_T | 0.9944 |
|  | dur_day_total_LIG_min | 6 | 934 | 248.47+-64.8_T | 232.55+-87.1_F | 0.1 |
|  | dur_day_total_MOD_min | 6 | -0.06 | 61.01+-29.1_T | 60.64+-30.2_T | 0.9519 |
|  | dur_day_total_VIG_min | 6 | 1338 | 0.32+-1.0_F | 0.56+-1.2_F | 0.1999 |
| Activity_type | time_sed | 6 | 694 | 0.45+-0.1_F | 0.47+-0.1_T | 0.572 |
|  | time_stand | 6 | 614 | 0.52+-0.2_F | 0.5+-0.1_T | 0.7431 |
|  | time_walk | 6 | 495 | 0.03+-0.0_F | 0.02+-0.0_F | 0.0944 |
| HR | mean | 6 | 1.82 | 75.51+-7.4_T | 79.07+-8.9_T | 0.0741 |
|  | sd | 6 | 0.59 | 12.66+-3.1_T | 13.2+-4.3_T | 0.5547 |
|  | min | 6 | 756 | 52.0+-10.5_F | 55.0+-9.0_F | 0.2047 |
|  | max | 6 | 707 | 158.0+-51.5_F | 164.03+-26.3_T | 0.475 |
| Resp | mean | 6 | 702 | 17.18+-2.9_F | 17.47+-1.3_T | 0.5122 |
|  | sd | 6 | 534 | 3.62+-0.7_F | 3.47+-0.4_T | 0.218 |
|  | min | 6 | 659 | 4.0+-1.0_F | 4.0+-2.0_F | 0.8466 |
|  | max | 6 | 677 | 36.0+-4.0_F | 36.64+-3.7_T | 0.7075 |
| Sleep | sleeponset | 6 | 894 | 22.53+-0.5_F | 22.39+-0.4_T | 0.053 |
|  | wakeup | 6 | 1.84 | 32.21+-0.5_T | 32.42+-0.6_T | 0.0692 |
|  | sleep_efficiency | 6 | -1.72 | 0.79+-0.1_T | 0.77+-0.1_T | 0.0884 |
|  | N_atleast5minwakenight | 6 | 1288.5 | 4.23+-1.9_T | 4.19+-2.7_F | 0.3564 |
|  | dur_spt_sleep_min | 6 | 0.4 | 450.27+-43.7_T | 453.94+-45.6_T | 0.6895 |
|  | dur_spt_wake_IN_min | 6 | 1322 | 88.88+-26.2_T | 97.44+-46.6_F | 0.2438 |
|  | dur_spt_wake_LIG_min | 6 | 1401 | 21.72+-14.1_F | 26.68+-18.3_F | 0.082 |
|  | dur_spt_wake_MOD_min | 6 | 1558 | 2.97+-2.3_F | 4.65+-4.8_F | 0.004 |
|  | dur_spt_wake_VIG_min | 6 | 1421 | 0.01+-0.0_F | 0.05+-0.1_F | 0.0564 |
|  | dur_spt_min | 6 | 2.33 | 568.87+-40.8_T | 587.94+-39.4_T | 0.0219 |
| Activity_intensity | dur_day_MVPA_bts_1_min | 7 | 815 | 23.07+-22.0_F | 13.82+-19.6_F | 0.3813 |
|  | dur_day_IN_bts_30_min | 7 | 1069 | 243.33+-135.5_F | 293.1+-111.3_T | 0.236 |
|  | dur_day_IN_bts_10_30_min | 7 | 830 | 103.13+-26.1_T | 98.36+-33.3_F | 0.4512 |
|  | dur_day_IN_bts_1_10_min | 7 | -1.15 | 117.59+-31.7_T | 108.77+-34.2_T | 0.2586 |
|  | dur_day_LIG_bts_10_min | 7 | 893 | 19.94+-28.9_F | 16.69+-24.0_F | 0.8101 |
|  | dur_day_LIG_bts_1_10_min | 7 | 815 | 91.07+-33.5_T | 89.72+-49.4_F | 0.3813 |
|  | dur_day_total_IN_min | 7 | 0.5 | 550.62+-72.3_T | 558.45+-66.7_T | 0.6192 |
|  | dur_day_total_LIG_min | 7 | 795 | 241.25+-64.9_T | 233.46+-75.2_F | 0.2991 |
|  | dur_day_total_MOD_min | 7 | -1.02 | 62.61+-30.2_T | 56.03+-27.4_T | 0.3123 |
|  | dur_day_total_VIG_min | 7 | 988.5 | 0.36+-1.2_F | 0.51+-1.1_F | 0.5965 |
| Activity_type | time_sed | 7 | 460 | 0.46+-0.2_F | 0.45+-0.1_T | 0.5827 |
|  | time_stand | 7 | 0.94 | 0.49+-0.2_T | 0.52+-0.1_T | 0.353 |
|  | time_walk | 7 | 433 | 0.03+-0.0_F | 0.02+-0.0_F | 0.3711 |
| HR | mean | 7 | 2.39 | 75.89+-8.4_T | 80.63+-7.0_T | 0.0219 |
|  | sd | 7 | 522 | 12.33+-4.3_F | 13.12+-3.7_T | 0.8181 |
|  | min | 7 | 581.5 | 53.0+-10.0_F | 55.0+-9.5_F | 0.3212 |
|  | max | 7 | 557.5 | 160.0+-47.0_F | 165.63+-24.3_T | 0.4929 |
| Resp | mean | 7 | 516 | 17.26+-2.4_F | 17.38+-1.5_T | 0.8781 |
|  | sd | 7 | 424 | 3.61+-0.6_F | 3.45+-0.4_T | 0.3128 |
|  | min | 7 | 498 | 4.0+-2.0_F | 4.0+-1.0_F | 0.9419 |
|  | max | 7 | 438.5 | 36.0+-6.0_F | 35.84+-3.1_T | 0.407 |
| Sleep | sleeponset | 7 | 739 | 22.5+-0.5_F | 22.37+-0.4_T | 0.135 |
|  | wakeup | 7 | -0.07 | 32.31+-0.5_T | 32.3+-0.6_T | 0.9473 |
|  | sleep_efficiency | 7 | -0.53 | 0.79+-0.1_T | 0.78+-0.1_T | 0.602 |
|  | N_atleast5minwakenight | 7 | -0.16 | 4.44+-2.0_T | 4.37+-1.9_T | 0.8707 |
|  | dur_spt_sleep_min | 7 | 0.36 | 450.84+-42.3_T | 454.8+-50.5_T | 0.7233 |
|  | dur_spt_wake_IN_min | 7 | 908 | 92.08+-39.7_F | 93.34+-33.2_T | 0.906 |
|  | dur_spt_wake_LIG_min | 7 | 1040 | 24.03+-14.4_F | 29.0+-13.6_T | 0.3427 |
|  | dur_spt_wake_MOD_min | 7 | 1115 | 3.2+-2.7_F | 4.38+-5.1_F | 0.1188 |
|  | dur_spt_wake_VIG_min | 7 | 1030.5 | 0.02+-0.1_F | 0.02+-0.1_F | 0.3776 |
|  | dur_spt_min | 7 | 0.99 | 574.99+-42.6_T | 583.7+-36.8_T | 0.3272 |
